# Reduction in Malaria Incidence following Indoor Residual Spraying with Actellic 300 CS in a Setting with Pyrethroid Resistance: Mutasa District, Zimbabwe

**DOI:** 10.1371/journal.pone.0151971

**Published:** 2016-03-28

**Authors:** Mufaro Kanyangarara, Edmore Mamini, Sungano Mharakurwa, Shungu Munyati, Lovemore Gwanzura, Tamaki Kobayashi, Timothy Shields, Luke C. Mullany, Susan Mutambu, Peter R. Mason, Frank C. Curriero, William J. Moss, Southern Africa International Centers of Excellence for Malaria Research

**Affiliations:** 1 Department of International Health, Johns Hopkins University Bloomberg School of Public Health, Baltimore, Maryland, United States of America; 2 Biomedical Research Training Institute, Harare, Zimbabwe; 3 Department of Medical Laboratory Sciences, University of Zimbabwe College of Health Sciences, Harare, Zimbabwe; 4 Department of Epidemiology, Johns Hopkins University Bloomberg School of Public Health, Baltimore, Maryland, United States of America; 5 National Institute of Health Research, Harare, Zimbabwe; National Institute for Communicable Diseases/NHLS, SOUTH AFRICA

## Abstract

**Background:**

More than half of malaria cases in Zimbabwe are concentrated in Manicaland Province, where seasonal malaria epidemics occur despite intensified control strategies. Recently, high levels of pyrethroid and carbamate resistance were detected in *Anopheles funestus*, the major malaria vector in eastern Zimbabwe. In response, a single round of indoor residual spraying (IRS) using pirimiphos-methyl (an organophosphate) was implemented in four high burden districts of Manicaland Province from November 1, 2014 to December 19, 2014. The objective of this study was to evaluate the effect of this programmatic switch in insecticides on malaria morbidity reported from health care facilities in Mutasa District, one of the worst affected districts in Manicaland Province.

**Methods:**

The number of weekly malaria cases for each health facility 24 months prior to the 2014 IRS campaign and in the subsequent high transmission season were obtained from passive case surveillance. Environmental variables were extracted from remote-sensing data sources and linked to each health care facility. Negative binomial regression was used to model the weekly number of malaria cases, adjusted for seasonality and environmental variables.

**Results:**

From December 2012 to May 2015, 124,206 malaria cases were reported from 42 health care facilities in Mutasa District. Based on a higher burden of malaria, 20 out of 31 municipal wards were sprayed in the district. Overall, 87.3% of target structures were sprayed and 92.1% of the target population protected. During the 6 months after the 2014 IRS campaign, a period when transmission would have otherwise peaked, the incidence of malaria was 38% lower than the preceding 24 months at health facilities in the sprayed wards.

**Conclusions:**

Pirimiphos-methyl had a measurable impact on malaria incidence and is an effective insecticide for the control of *An*. *funestus* in eastern Zimbabwe.

## Introduction

Malaria is a major public health problem in Zimbabwe, which lies in the southern fringe of malaria transmission in sub-Saharan Africa [1]. Approximately half of the population of 12.9 million is at risk for malaria [2, 3]. During 2013, there were 377,872 cases and 351 deaths attributed to malaria, with the greatest burden among children younger than five years of age, pregnant women and people living with HIV/AIDS [3–5]. *Plasmodium falciparum* accounts for 98% of all reported malaria cases and *Anopheles arabiensis* is the major malaria vector in much of the country. The epidemiological pattern of malaria transmission varies spatially and temporally in Zimbabwe, and is largely driven by elevation and rainfall patterns [5, 6]. The rainy season spans November to April, while peak malaria transmission usually occurs between February and May as a result of the preceding rains.

Malaria control in Zimbabwe relies on case management, insecticide-treated nets (ITNs) and indoor residual spraying (IRS), which have successfully reduced malaria transmission in many parts of the country [3, 7]. However, the success of the malaria control program has been challenged by resurgence, particularly in Manicaland Province that continues to have a high burden of malaria. In 2013, Manicaland Province accounted for 51% of the malaria morbidity and 35% of the malaria mortality burden, despite encompassing less than 14% of the national population [2, 3]. One potential reason for the resurgence of malaria is the development of insecticide resistance in *An*. *funestus*, the major malaria vector in the region [8–10]. Recent (2013–14) insecticide resistance monitoring in Mutasa District using standard World Health Organization (WHO) testing methods showed that *An*. *funestus* from Manicaland was highly resistant to pyrethroids and carbamates [11]. Vector susceptibility tests conducted with organochlorines (DDT and dieldrin) and organophosphates (malathion, fenitrothion and pirimiphos-methyl) showed 100% mortality 24 hours post-exposure [11], suggesting that a change in IRS strategy may be more effective. Insecticide resistance has important implications for malaria control because pyrethroids have been the primary insecticides used by the Zimbabwe National Malaria Control Program (NMCP) for IRS.

In response to the emerging insecticide resistance, the President’s Malaria Initiative (PMI) through the United States Agency for International Development (USAID) and in collaboration with the Ministry of Health and Child Care, switched insecticide classes for IRS from pyrethroids to an organophosphate in four high transmission districts in Manicaland Province (Chimanimani, Mutasa, Mutare and Nyanga) during the 2014 IRS campaign. The goal of the present study was to evaluate the population-level impact of the switch to an organophosphate insecticide on malaria morbidity in Mutasa District. The underlying study hypothesis was that IRS using an organophosphate insecticide would result in a reduction in malaria case incidence at health facilities in sprayed areas during the subsequent high transmission season (December 2014–May 2015), adjusting for environmental and climatic variables that could impact malaria transmission.

## Methods

### Study Area

Mutasa District is situated in the north-east of Zimbabwe bordering Mozambique, and encompasses an area of 622 km^2^ that stretches from 18.20° to 18.58°S latitude and from 32.71° to 33.06° E longitude. Elevation rises from 600 meters in the valleys to 2,500 meters in the mountains. The human population was estimated to be 169,756 residents representing 42,479 households at the time of the 2012 census [2]. The district is irrigated by two major rivers, the Honde and the Pungwe, and the major economic activity is agriculture. The average daily temperature is 21.5°C; November is the hottest month with an average daily temperature of 24.5°C and July is the coolest month with an average daily temperature of 16.3°C. Malaria transmission is characterized as seasonal and unstable with major outbreaks during the rainy season which usually runs from November to April each year. In 2014, Mutasa District received 2,352 millimeters of rainfall between November and April and 96 millimeters during the dry season as measured at the Southern Africa ICEMR station in Hauna, the main town in the lower valley. The population covered by pyrethroid-based IRS in Mutasa District was 88% in 2012 and 91% in 2013. ITNs were not distributed in 2012 but were distributed to the general population in 2013 and to boarding schools in 2014. ITN distribution increased household ITN ownership from 64% to 87% between 2012 and 2013. Reported ITN usage averaged 52% during the study period [12].

### Indoor Residual Spraying with Pirimiphos-Methyl

**Fig 1:**
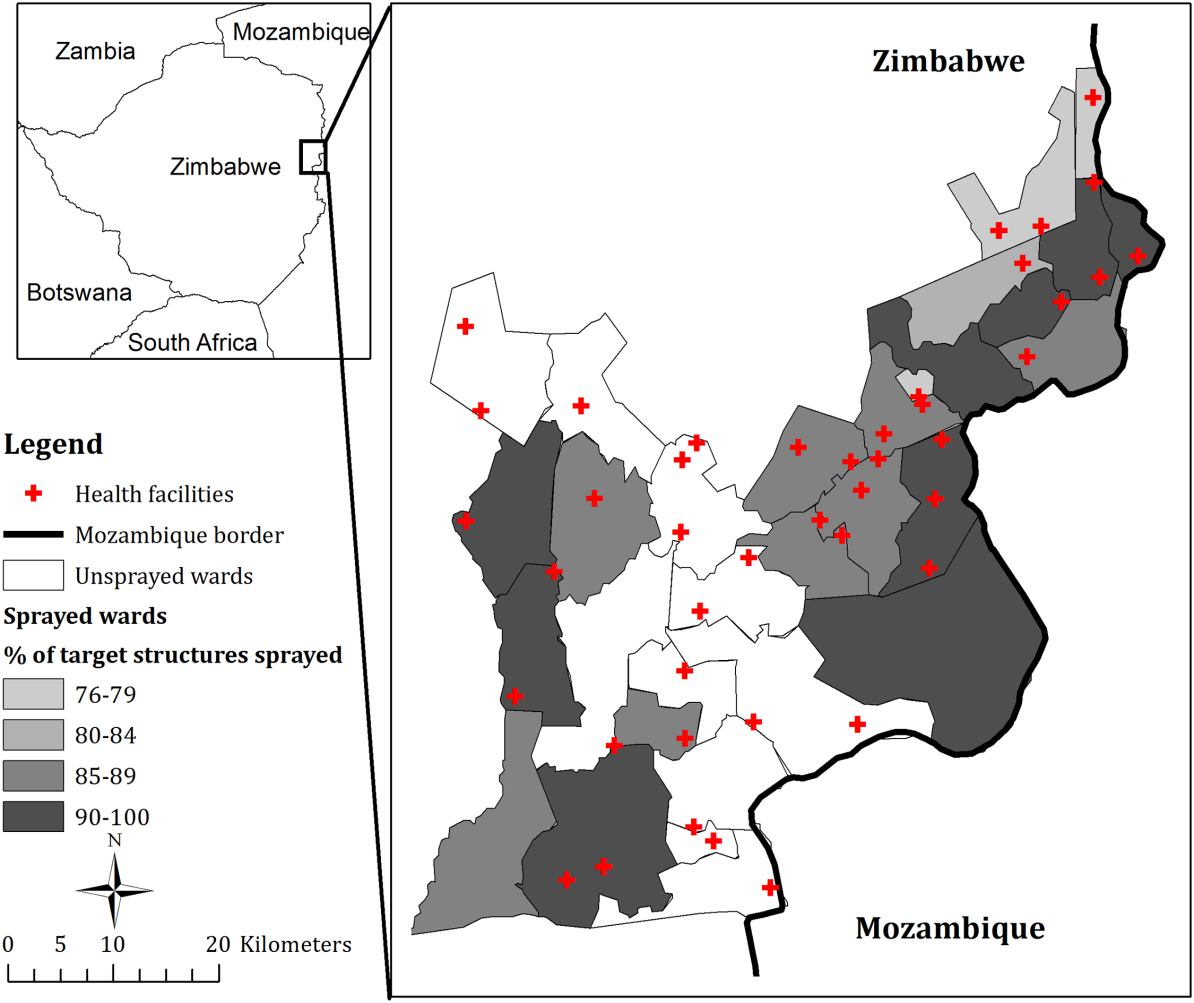
Geographic Distribution of Health Facilities in Mutasa District, Zimbabwe.

An organophosphate insecticide, pirimiphos-methyl (Acetellic 300 CS, Syngenta, Sweden), was selected for the 2014 IRS campaign in Mutasa District. Trained and experienced spray operators conducted the IRS operations between November 1, 2014 and December 19, 2014. The spraying was done to attain the recommended dose for malaria control of 1.0 g active ingredient/m^2^ [13]. Each of the wards in the district encompasses one or more entire health facility catchment areas. From among the district’s 31 municipal wards, 20 malaria-prone wards were selected, which included 43,103 structures and covered a population of 87,275 (Fig 1). According to PMI, 87.3% of targeted structures were found and sprayed, while 92.1% of the target population resided in houses sprayed [14].

### Malaria Case Data

The health management information system (HMIS) of the Ministry of Health and Child Care routinely collects weekly malaria-related data at the health-facility level in Mutasa District. Indicators include the number of positive rapid diagnostic tests (RDT) using SD Bioline Ag. Pf., patients clinically diagnosed with malaria, and malaria deaths, stratified by age (< 5 years and ≥ 5 years). This passive surveillance system has previously been used to monitor secular trends in malaria morbidity and assess the impact of malaria interventions [15]. The system is operational at 43 health care facilities offering malaria diagnosis and treatment. Two-thirds of these facilities are government funded. Geographic coordinates of the health care facilities and the size of catchment area population for each health care facility were acquired through the district health team or field visits. Catchment area population size was adjusted for population growth by assuming linear growth during monthly intervals, summing to an annual population growth of 4% (as projected by the 2012 census) [2].

The primary dependent variable was the weekly number of malaria cases confirmed by RDT at each health care facility. Data prior to the completion of the 2014 IRS campaign included 105 epidemiological weeks (2012 week 50–2014 week 50) and data following the completion of the 2014 IRS campaign included 24 weeks (2014 week 51–2015 week 22). Weekly confirmed malaria case counts at each health care facility were standardized as rates per 1,000 population using estimates of the population within each health care facility catchment area.

### Primary Exposure Variables

To assess whether weekly malaria incidence decreased following the IRS campaign and whether this decrease exceeded that which might be expected on the basis of trends prior to the 2014 IRS campaign two binary variables were created: time period indicating before and after the completion of the 2014 IRS campaign (pre vs. post IRS) and spray status indicating whether a health facility was in the sprayed zone (sprayed versus unsprayed). The period variable estimates the difference in malaria morbidity between the pre- and post- IRS campaign (an overall IRS effect) and the spray status variable estimates the difference in malaria morbidity for health facilities located in the sprayed versus unsprayed zones (assessing/controlling for geographic trends). The period by spray status interaction allows the model to estimate a more specific IRS effect of whether there was a difference in malaria morbidity before and after the IRS campaign for health facilities in the sprayed zones.

### Potential Confounding Variables

To account for seasonal variations in malaria cases, indicator variables of the calendar month in which the case was reported were introduced into the model. Additionally, to account for year-to-year variation of malaria cases, indicator variables representing the years 2012, 2013 and 2014 also were entered into the model (2015 was the reference year).

Several environmental variables that affect the survival and reproduction of the malaria vector and the development, survival, and reproduction rates of the *Plasmodium* within *Anopheles* were included as potential explanatory variables. Elevation, rainfall estimate (RFE), day and night land surface temperature (LST) and Normalized Difference Vegetation Index (NDVI) were extracted from remote-sensing sources for each health care facility. Elevation data was estimated at 90-meter resolution from the Shuttle Radar Topography Mission (SRTM) digital elevation model. Decadal (10 day) RFE data were downloaded via the Africa Data Dissemination Service (ADDS). RFE is an estimation of rainfall from the Meteosat 7 satellite that has been calibrated against ground-based rain gauge data. Day and night LST and NDVI were obtained from Moderate Resolution Imaging Spectroradiometer (MODIS) sensor aboard the NASA satellites Aqua and Terra. Daily day and night LST data, expressed as °C were extracted from MOD11A products with 0.25 km by 0.25 km spatial resolution. LST is a proxy for the prevailing temperature of the air. Sixteen-day composite NDVI was extracted from MOD13Q1 products with 1 km by 1 km spatial resolution. NDVI is correlated with the amount of vegetation and typically ranges from -1 to 1. Values close to 1 indicate very dense vegetation, while values zero and below represent water or impervious land forms.

Hydrologic analysis was performed using the digital elevation model to create a stream network layer, containing attribute information expressing the classifications of streams using Strahler’s method [16]. In this classification, a stream of order 2 is formed when two streams of order 1 join. Stream classifications ranged from 1 indicating low volume streams typically present only during the rainy season, to 4 indicating high volume, year-round streams usually found at lower elevations. The two major rivers in Mutasa District, the Pungwe and Honde, had a stream order of 4. The Euclidean distance from each household to the nearest stream in each of the 4 classes was calculated in ArcGIS 10.2 (ESRI, Redlands, California).

The time unit of analysis was one week. Parasitological data were collected on a weekly basis, while environmental data obtained from remote sensing were obtained at different temporal resolutions. Consequently, all time-varying environmental data were rectified into weekly values by taking averages of daily values and disaggregating decadal values. Due to cloud cover and satellite malfunctions, day and night LST were missing for 0.36% and 0.33% of daily values, respectively. Missing values were imputed by assuming a linear trend for non-missing data. In other words, the difference between two succeeding data points was assumed to be equally distributed among the times with no observed value in between. To account for possible elapsing (lag) time in the effect of time-varying environmental variables on the outcome (weekly number of malaria cases confirmed by RDT), lags up to 3 months were incorporated. Three months was chosen as the maximum biologically plausible lag between malaria incidence and environmental variables.

### Statistical Analysis

Descriptive statistics were used to examine the characteristics of the sample prior to and following the 2014 IRS campaign. Rather than conduct facility-level analyses for each of the health care facilities, a negative binomial panel data model was run at the district-level in which the dependent variable was the number of malaria cases by health care facility and week. The number of weekly malaria cases confirmed by RDT for a health care facility was modeled by using a negative binomial regression model for all facilities with catchment area population size as the regression offset to model the rate of RDT positivity. Poisson regression models for the RDT confirmed cases were consistently over-dispersed (greater variation in the data than the Poisson model can accommodate) throughout the analysis and hence the model was replaced with negative binomial regression, which allows for a scaling factor on the model variance to account for over-dispersion. Further, regression inference was based on a generalized estimating equations (GEE) approach to account for the within health facility repeated measures correlation [17].

All variables were introduced separately as independent variables in the univariate adjusted regression models, adjusted for seasonality and health facility. Variables with a p***-***value <0.1 in the univariate adjusted regression models were considered as potential candidates for the multivariable selection process. A manual stepwise backwards elimination approach was used to select environmental variables and their associated lags. Selection of the model with the best fit, the best working correlation structure in GEE and optimal lag sizes for time-varying environmental factors were determined by comparing quasi-likelihood under the independence model criterion (QIC) values of different models. The QIC is a modification of the Akaike Information Criterion (AIC) for GEE models and similar to the AIC, a model that minimizes the QIC is considered the best fit [18]. Results were expressed in terms of incidence risk ratios (IRR) with corresponding 95% confidence intervals to quantify the expected change in the incidence rate of malaria when the exposure variable was positive or increasing. A p-value <0.05 was considered statistically significant. The root mean square error (RMSE) standardized by catchment area population was used to evaluate the agreement between observed and model predicted number malaria cases for the district. The RMSE per 1,000 catchment area population was also calculated by health care facility to determine differences in predictions by facility.

To assess the impact of the 2014 IRS campaign over time on model predictions, sensitivity analyses were conducted varying the cutoff point dividing the time from the week IRS began to the week IRS was completed. The optimal breakpoint was considered the point (week) where the QIC was smallest. Sensitivity analyses were also performed using data aggregated to a month to investigate whether the results were dependent on the time unit of analysis. Environmental data were linked to the health care facility reports mapped in ArcGIS 10.2 (Redlands, California). All statistical analyses were conducted in STATA 11.2 (College Station, Texas).

### Ethical Considerations

The Institutional Review Boards of the Johns Hopkins Bloomberg School of Public Health, the Biomedical Research and Training Institute and the Medical Research Council of Zimbabwe approved this research. The analysis was based on malaria reports collected routinely by the NMCP in Zimbabwe. It was not necessary or possible to obtain written informed consent as these reports were de-identified and anonymized prior to aggregation.

## Results

After excluding one health care facility that started reporting data in April 2013, the analytical sample comprised 42 health facilities that had complete data on the weekly number of malaria cases from December 2012 to May 2015. The 20 malaria-prone wards selected for spraying covered the catchment areas of 28 health facilities, while the remaining 11 wards covered the catchment areas of 14 health facilities. The campaign succeeded in attaining the project goal of spraying 85% of target structures [14], and spray coverage by ward ranged from 76% to 96% (Fig 1).

During the study period, 124,206 malaria cases were reported, of which 113,208 were from health facilities in sprayed wards. During the two high transmission seasons preceding the IRS campaign, the number of malaria cases reported averaged 42,586 compared to 12,222 malaria cases in the high transmission season following application of the organophosphate. The malaria incidence rate fell from 270 per 1,000 in the two high transmission seasons pre-IRS to 71 per 1,000 post-IRS (Table 1), representing a crude incidence risk ratio of 0.26.

**Table 1:** Characteristics of sprayed and unsprayed wards before and after the 2014 IRS campaign in Mutasa District, Zimbabwe.

	Pre-IRS^a^		Post-IRS
	High transmission season	Low transmission season	High transmission season
**Total number of malaria cases reported**			
Sprayed wards	42,586	8,612	10,813
Unsprayed wards	4,232	563	1,409
All wards	46,818	9,175	12,222
**Incidence rate (per 1,000 individuals)**			
Sprayed wards	362	73	92
Unsprayed wards	76	10	25
All wards	270	53	71

^a^ Pre-IRS values are averaged for the two preceding years

Several significant associations were identified between malaria incidence and environmental variables in both the univariate and multivariate negative binomial regression models (Table 2). After adjustment, an increase of 10 millimeters in RFE (estimated rainfall) resulted in a 2% increase in malaria incidence 6 weeks later (IRR 1.02, 95% CI 1.01–1.03). Each 1°C increase in night LST at a 10-week lag resulted in a 2% increase in malaria incidence (IRR 1.02, 95% CI 1.01–1.04). In contrast, each 1°C increase in day LST at a 1-week lag resulted in a 2% reduction in malaria incidence (IRR 0.98, 95% CI 0.97–0.99). Malaria incidence decreased with increasing elevation; every 100-meter increase in elevation was associated with a 20% reduction in malaria (IRR 0.80, 95% CI 0.73–0.88). A similar reduction in malaria incidence was found for every one kilometer increase in distance from a second order stream (IRR 0.81, 95% CI 0.76–0.86) (Table 2).

**Table 2:** Univariate and multivariate incidence rate ratios for reported malaria cases from negative binomial regression.

	Adjusted Univariate			Multivariate^a^		
	IRR	95% CI	p	IRR	95% CI	p
Intervention period						
Pre-IRS	1.00			1.00		
Post-IRS	0.78	0.63–0.98	0.03	1.02	0.74–1.41	0.9
Spray status						
Unsprayed	1.00			1.00		
Sprayed	15.8	15.44–16.20	<0.001	0.94	0.65–1.34	0.7
Interaction: Spray status by intervention period	0.53	0.41–0.69	<0.001	0.62	0.51–0.76	<0.001
Elevation (per 100 meters)	0.83	0.70–0.99	0.04	0.80	0.76–0.83	<0.001
RFE at a 6 week lag (per 10 mm)	1.01	1.00–1.02	0.03	1.02	1.01–1.04	0.004
Day LST at a 1 week lag (per 1°C)	0.98	0.97–0.99	<0.001	0.98	0.97–0.99	<0.001
Night LST at a 10 week lag (per 1°C)	1.03	1.02–1.04	0.004	1.03	1.01–1.05	<0.001
Distance to second order streams (per 1 kilometer)	0.73	0.69–0.77	0.02	0.81	0.76–0.86	<0.001

^a^ Adjusted for season (month and year indicators) and health facility, with an offset of log of population size and an autoregressive correlations structure specified.

Adjusting for annual and seasonal trends, environmental variables and clustering at health care facilities, there were no significant differences in malaria incidence between health facilities in sprayed wards compared to unsprayed wards prior to the 2014 IRS campaign (IRR 0.94, 95% CI 0.65–1.34). However, there was a 38% decline in predicted malaria cases after the 2014 IRS campaign in sprayed wards compared to before (IRR 0.62, 95% CI 0.51–0.76). In contrast, no change in predicted malaria incidence was observed after the 2014 IRS campaign compared to before in health facilities located in unsprayed wards (IRR 1.02, 95% CI 0.74–1.41) (Table 2).

**Fig 2:**
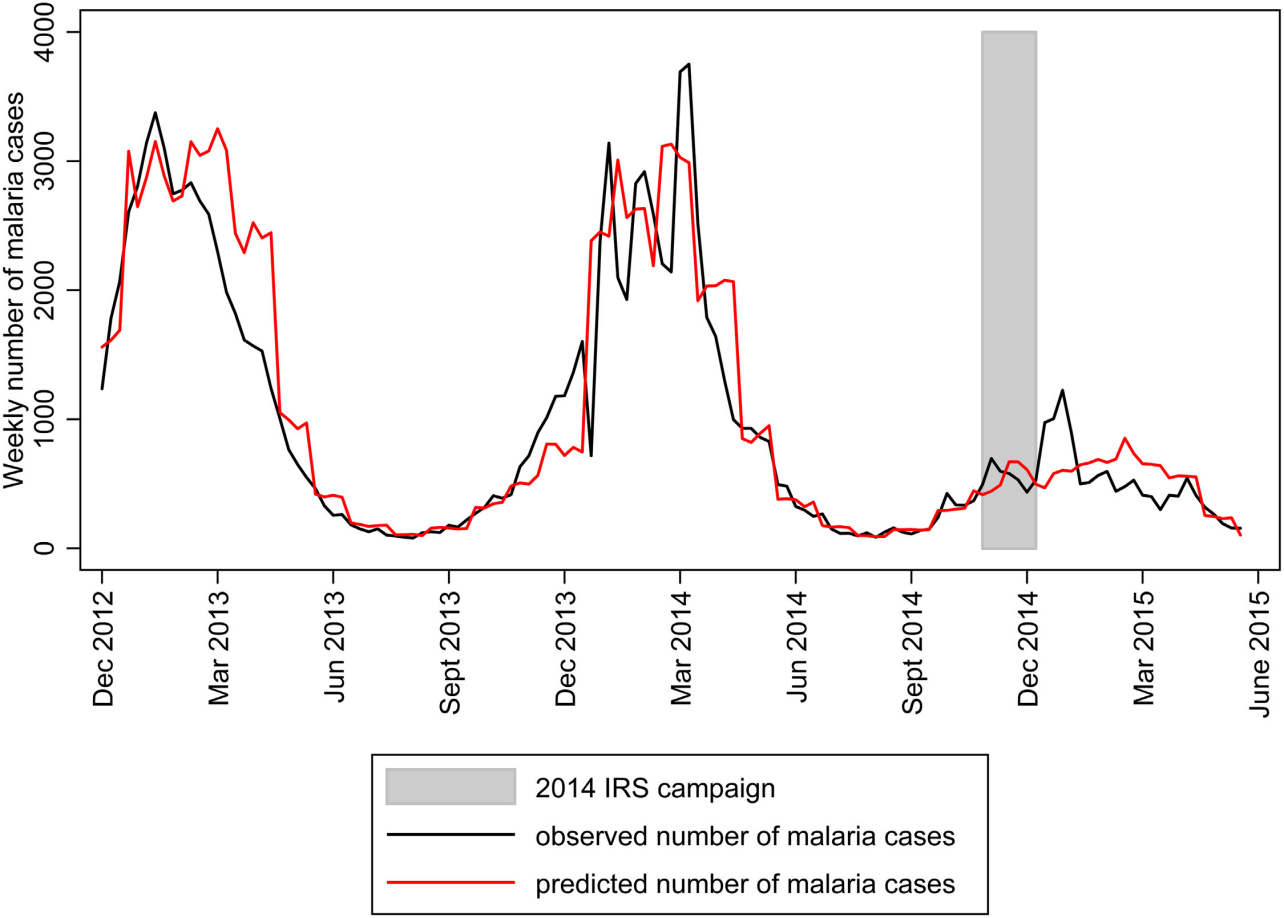
Observed and predicted weekly malaria cases in Mutasa District, 2012–2015.

The observed weekly cases for the study area agreed closely with the predicted counts throughout the study period (Fig 2). For the entire study period, the final model predicted 132,458 cases across all 42 health facilities, whereas, 124,206 cases were reported. However, there was variability by health facility in how closely the predicted and observed total number of cases were matched. The observed number of malaria cases was higher than expected given the surrounding environmental conditions and seasonal variations at three health facilities (Old Mutare, Honde Mission, and Hauna). The model also over-predicted the number of malaria cases given the surrounding environmental conditions and seasonal variations in some health facilities (Zongoro, Dreaanane and Gatsi). The RMSE per 1,000 catchment areas population was poor for St Peter’s, Sagambe, and EHPL health facilities and good for Mutasa, Samanga and Bonda health facilities (S1 Table).

The model with first order autoregressive (AR1) correlation was found to fit the data reasonably well compared to other choices of a working correlation matrix. The fit of the final model was assessed using the QIC and RMSE. The addition of environmental variables to the model improved the model fit and predictions, as the QIC was lower for the final model than the null model (QIC for the null 17,867 versus QIC for the full model 3,934). Minimal differences were identified after assessing the sensitivity of the findings to different cut-offs for the binary variable indicating time before and after the 2014 IRS campaign, suggesting the results were robust to the cut-off used. Furthermore, sensitivity analyses consisting of data aggregated to the month level produced similar estimates of the effectiveness of the IRS campaign.

## Discussion

Owing to insecticide resistance in *An*. *funestus* in eastern Zimbabwe, the NMCP with support from PMI began a large-scale IRS campaign with the organophosphate pirimiphos-methyl in four high transmission districts in Manicaland Province, including Chimanimani, Mutare, Mutasa and Nyanga Districts. Using health facility surveillance data, a reduction in the incidence of malaria was observed in one of the four high transmission districts. During the subsequent high transmission season following the switch from pyrethroids to organophosphates, a 38% decline in malaria incidence was reported by health care facilities from wards that conducted IRS after accounting for possible confounding by environmental and climatic variables. Previous research demonstrated that switching to an organophosphate insecticide for IRS effectively reduced biting rates and vector densities in areas with pyrethroid-resistant vectors in Ghana, Benin, Tanzania and Zambia [19–23]. This study demonstrates a reduction in malaria morbidity using health care facility surveillance data.

Malaria transmission was associated with rainfall, proximity to second order streams, elevation and temperature. These results concur with previous studies that found that elevation [5], temperature, rainfall [6, 24] and proximity to streams [25, 26] were associated with malaria risk. Zimbabwe has experienced periodic droughts that could impact malaria transmission and confound interpretation of the association between malaria control interventions and outcomes. After adjusting for climatic variables and seasonality, malaria incidence rates decreased following the 2014 IRS campaign, supporting the conclusion that switching to organophosphates in this setting contributed to the observed reduction in malaria morbidity. No major political, socio-economic or health-care changes with the potential to reduce malaria morbidity by almost half occurred in Mutasa District during the study period.

Typically, data from health care facilities only includes data on the number of suspected malaria cases. However, the HMIS in Zimbabwe allows reports of confirmed malaria cases. In calculating incidence rates, the denominator used was the estimated catchment area population size. The reliability of this value has been questioned as this assumes that individuals visit the closest health facility in their catchment area. However, the main results did not change after including an offset for catchment area population size, indicating that the reported catchment area population size may be a reliable estimate. The study also underscores the utility of HMIS data in the evaluation of population level interventions. The HMIS has the advantage of providing quality data quickly and easily, with minimal additional investment. Additionally, HMIS reflects the burden of disease on the health system. Results from this study further suggest that passive surveillance data from the HMIS in Zimbabwe was sufficiently sensitive, and the effect size sufficiently large, to detect a reduction in malaria morbidity following the 2014 IRS campaign.

There are several important limitations to this analysis. Causal inferences between IRS and reduction in malaria incidence should be made with caution as spraying was not implemented as part of a randomized control trial. However, data from 14 health facilities located in unsprayed wards were included in the analysis to serve as a comparison group and better estimate secular changes in malaria morbidity unassociated with the 2014 IRS campaign, particularly changes in rainfall. Although the univariate model indicated that health care facilities in unsprayed wards had a lower burden of malaria, the multivariable model showed no significant differences between health care facilities in sprayed and unsprayed wards prior to the 2014 IRS campaign, suggesting that environmental and climatic variables adequately adjusted for the differences. However, the analyses did not account for other factors such as population movement, changes in health seeking behaviors or changes in ITN coverage that also could impact malaria incidence. This seems reasonable given that the rural population of Mutasa District is relatively stable, with access to health facilities providing malaria diagnosis and treatment. Following the distribution of ITNs in 2013, household surveys recorded an increase in household ITN ownership but no changes in ITN usage (unpublished data); therefore, it is unlikely the ITN distribution in 2013 affected the estimate of the reduction in malaria incidence. Additionally, although the number of suspected malaria cases was not explicitly modeled, a descriptive analysis did not indicate changes in diagnostic practices over the study period (data not shown). The HMIS in Zimbabwe has been in place for decades and was previously used to evaluate the impact of changes in malaria morbidity [15], construct empirical seasonality maps [23] and describe the spatial and temporal distribution of malaria [27, 28].

The pronounced decline in malaria morbidity observed is evidence supporting the benefit of pirimiphos-methyl in an area with high levels of pyrethroid resistance and with high coverage in the targeted districts. Although the IRS strategy was successful, continued entomological monitoring for insecticide resistance will be necessary. With emerging resistance to multiple insecticides, novel strategies to manage insecticide resistance need to be developed.

## Supporting Information

S1 Table Summary of prediction accuracy for the study period for the final model across 42 health facilities. (DOCX)
