# Supplementary material for: Reduction in Malaria Incidence following Indoor Residual Spraying with Actellic 300 CS in a Setting with Pyrethroid Resistance: Mutasa District, Zimbabwe
Source: PLoS One. 2016 Mar 28;11(3):e0151971. doi: 10.1371/journal.pone.0151971 (PMC4809594; doi:10.1371/journal.pone.0151971)
Supplement: S1 Table — (DOCX) [file pone.0151971.s001.docx]

| Health facility | Observed number of malaria cases | Predicted number of malaria cases | Difference (%) | RMSE | Catchment area population | RMSE per 1,000 catchment area population |
| --- | --- | --- | --- | --- | --- | --- |
| Bonda | 229 | 302 | 32 | 2 | 4,595 | 0.53 |
| Chinamasa | 107 | 113 | 6 | 1 | 1,151 | 1.08 |
| Chisuko | 13,447 | 21,722 | 62 | 133 | 9,681 | 13.75 |
| Chitombo | 1,003 | 808 | -19 | 8 | 4,106 | 1.90 |
| Dreaanane | 48 | 82 | 71 | 1 | 980 | 0.69 |
| EHPL | 7,553 | 8,265 | 9 | 38 | 2,671 | 14.06 |
| Gatsi | 4,109 | 6,934 | 69 | 36 | 6,797 | 5.36 |
| Guta | 409 | 401 | -2 | 4 | 4,475 | 0.91 |
| Haparari | 230 | 257 | 12 | 2 | 2,660 | 0.86 |
| Hauna Clinic | 10,978 | 6,260 | -43 | 75 | 6,394 | 11.79 |
| Hauna Hospital | 5,776 | 5,337 | -8 | 36 | 5,198 | 6.94 |
| Honde Mission | 912 | 697 | -24 | 7 | 1,809 | 3.90 |
| Imbeza | 307 | 246 | -20 | 5 | 3,685 | 1.40 |
| Jombe | 716 | 677 | -5 | 7 | 3,736 | 2.00 |
| Katiyo | 3,563 | 4,132 | 16 | 19 | 2,126 | 8.78 |
| Mandeya II | 10,217 | 10,724 | 5 | 41 | 5,322 | 7.68 |
| Mapara | 181 | 170 | -6 | 4 | 1,324 | 2.90 |
| Moyoweshumba | 446 | 486 | 9 | 4 | 3,251 | 1.33 |
| Mt. Jeya | 1,021 | 1,164 | 14 | 7 | 3,165 | 2.17 |
| Mupotedzi | 1,582 | 1,491 | -6 | 10 | 3,590 | 2.80 |
| Mutasa | 275 | 256 | -7 | 2 | 5,175 | 0.37 |
| Ngarura | 5,455 | 5,319 | -2 | 22 | 2,845 | 7.69 |
| Old Mutare | 1,097 | 659 | -40 | 18 | 10,046 | 1.79 |
| Premier | 1,932 | 1,639 | -15 | 15 | 1,814 | 8.23 |
| Red Wing | 576 | 500 | -13 | 6 | 9,551 | 0.63 |
| Rupinda | 1,283 | 1,323 | 3 | 7 | 2,845 | 2.62 |
| Sadziwa | 189 | 236 | 25 | 4 | 1,621 | 2.58 |
| Sagambe | 12,220 | 10,255 | -16 | 55 | 2,683 | 20.49 |
| Sahumani | 1,779 | 1,645 | -8 | 11 | 3,031 | 3.63 |
| Sakupwanya | 538 | 522 | -3 | 4 | 5,156 | 0.78 |
| Samanga | 247 | 341 | 38 | 2 | 4,789 | 0.48 |
| Samaringa | 1,137 | 1,043 | -8 | 6 | 2,064 | 3.06 |
| Selbome | 221 | 224 | 1 | 3 | 1,950 | 1.41 |
| Sheba | 77 | 59 | -23 | 1 | 882 | 1.23 |
| Sherukuru | 559 | 452 | -19 | 6 | 2,294 | 2.83 |
| St. Augustine’s | 995 | 933 | -6 | 8 | 5,796 | 1.32 |
| St. Barbara’s | 168 | 185 | 10 | 2 | 2,093 | 1.01 |
| St. Peter’s | 20,589 | 26,121 | 27 | 141 | 6,141 | 22.90 |
| Triashill | 112 | 122 | 9 | 2 | 2,110 | 0.81 |
| Tsonzo | 1,011 | 1,264 | 25 | 14 | 10,567 | 1.34 |
| Zindi | 9,335 | 7,596 | -19 | 55 | 9,692 | 5.63 |
| Zongoro | 1,311 | 2,801 | 114 | 20 | 3,302 | 6.09 |
